# German guidelines on the diagnosis and treatment of neurosyphilis

**DOI:** 10.1186/s42466-020-00081-1

**Published:** 2020-11-17

**Authors:** Matthias Klein, Klemens Angstwurm, Stefan Esser, Kathrin Hahn, Matthias Maschke, Simone Scheithauer, Helmut Schoefer, Matthias Sturzenegger, Brigitte Wildemann, Jörg Weber

**Affiliations:** 1 Department of Neurology, LMU Klinikum Muenchen, Marchioninistr. 15, 81377 Munich, Germany; 2 Department of Neurology, Universitaetsklinik Regensburg, Universitaetsstr. 84, 93042 Regensburg, Germany; 3 Department of Dermatology, Universitaetsklinikum Essen, Hufelandstrasse 55, 45147 Essen, Germany; 4 Department of Neurology, Charite Berlin, Chariteplatz 1, 10117 Berlin, Germany; 5 Department of Neurology, Krankenhaus der Barmherzigen Brueder Trier, Nordallee 1, 54292 Trier, Germany; 6 Institute for Hygiene and Infectiology, Universitaet Goettingen, Robert-Koch-Str. 40, 37075 Göttingen, Germany; 7 Dr. Horst Schmidt Kliniken, Aukamm-Allee 33, 65191 Wiesbaden, Germany; 8 Department of Neurology, Inselspital Bern, Freiburgstrasse 15, 3010 Bern, Switzerland; 9 Department of Neurology, Universitaetsklinik Heidelberg, Im Neuenheimer Feld 672, 69120 Heidelberg, Germany; 10 Department of Neurology, Klinikum Klagenfurt, Feschnigstraße 11, 9020 Klagenfurt am Wörthsee, Austria

## Abstract

**Introduction:**

In view of the importance of neurosyphilis and the difficulties encountered in diagnosing it, the S1 guideline “Neurosyphilis” has been published by the German Society for Neurology (DGN) in accordance with the stipulations of the Association of the Scientific Medical Societies in Germany (AWMF). The present article is an abridged translation of that German guideline.

**Main recommendations:**

(a) Neurosyphilis can manifest as early neurosyphilis (meningitis, meningovascular neurosyphilis or syphilitic gummas) or late neurosyphilis (tabes dorsalis, general paresis). (b) The following diagnostic criteria help to establish the presence of probable neurosyphilis (always point iv, accompanied by any two of points i to iii): (i) subacute or chronic neuro-psychiatric symptoms; (ii) increased cerebrospinal fluid (CSF) cell count or signs of blood–CSF barrier disruption; (iii) positive effect of anti-neurosyphilis antibiotic therapy on clinical course and CSF findings; (iv) positive TPHA/TPPA or FTA test in serum. (c) The diagnosis of neurosyphilis is confirmed by the subsequent detection of intrathecal production of antibodies against *Treponema pallidum*. (d) In neurosyphilis, treatment with intravenous penicillin or ceftriaxone for 14 days is recommended. (e) The following parameters can be used to assess a therapeutic effect: clinical findings, serum VDRL, and CSF cell count.

**Conclusion:**

The German guideline on the diagnosis and treatment of neurosyphilis is a practical tool to support clinicians in diagnosing and treating patients with neurosyphilis. This article is an abridged translation of this guideline (Klein MW, J.; Angstwurm, K.; Esser, S.; Hahn, K.; Matschke, M.; Scheithauer, S.; Schoefer, H.; Sturzenegger, M.; Wildemann, B. Neurosyphilis, S1-Leitlinie. Deutsche Gesellschaft für Neurologie, Leitlinien für Diagnostik und Thearpie in der Neurologie 2020).

## Introduction

This article is an abridged translation of the German guideline on the diagnosis and therapy of neurosyphilis [1].

Syphilis is an infectious disease caused by *Treponema pallidum*, a Gram-negative bacterium of the Spirochaetaceae family. It is almost exclusively sexually transmitted. Around one third of the infected patients develop clinical signs of infection. Syphilis occurs in several stages [2, 3]. After a regional infection at the entry point (primary syphilis), a chronic recurrent disease can develop (secondary syphilis), with variable manifestations, followed by a latency phase lasting from several months to several years. A later inflammatory reaction against the pathogen can develop from the latency phase, characterized by granulomatous reactions (tertiary syphilis). The point in time at which so-called early syphilis changes to the latency phase, thus becoming late syphilis, is not defined consistently in the literature: The WHO defines early syphilis as the presence of symptoms over a maximum period of 2 years; European guidelines, the guidelines of the Centers of Disease Control and Prevention (CDC) and the Association of the Scientific Medical Societies in Germany (AWMF) S2k guideline “Diagnostik und Therapie von Syphilis” (“Diagnosis and treatment of syphilis”) define disease lasting 1 year or more as late syphilis [4, 5].

Neurological manifestations can occur at any stage after primary syphilis. A distinction is made between early neurosyphilis, occurring a few months to years after infection, and late neurosyphilis, arising years to decades after infection.

## Methods of guideline development

AWMF registry number 030/101 [1]. Level of guideline: S1. Date of Last Update: May 2020. Valid until April 30th, 2025. Edited by the German Neurological Society (Deutsche Gesellschaft für Neurologie, DGN). The guideline was approved by the guideline commission of the DGN, has been approved by the DGN and has been published in an extended version on the AWMF Guidelines repository [1]. Joint recommendation: The following societies participated in the guideline development and approved the final version: The following societies have approved the guideline: German Neurological Society (DGN), Swiss Neurological Society (Schweizerische Neurologische Gesellschaft), the Deutsche Gesellschaft für Liquordiagnostik und klinische Neurochemie (DGLN), the Austrian Neurological Society (Österreichische Gesellschaft für Neurologie), the German Society for Neuro-Aids and Neurological Infectious Diseases (Deutsche Gesellschaft für Neuro-Aids und Neuro-Infektiologie), the German AIDS Society (Deutsche AIDS Gesellschaft), the German STI Society (Deutsche STI Gesellschaft), and the German Dermatological Society (Deutsche dermatologische Gesellschaft).

## Epidemiology

Each year, around 5.6 million patients worldwide develop syphilis, with particularly high incidence in Africa [6]. According to the registry data of the Robert Koch Institute (RKI), the incidence of syphilis in Germany has been declining for many years and reached its lowest level in the late 1990s, with 1.4 cases reported per 100,000 inhabitants [7]. However, the number of new infections has risen continuously since 2010. In 2018, 7332 cases of syphilis were reported to the RKI (compared with 2716 reports in 2009). Men, especially men who have sex with men (MSM) are particularly at risk. This group accounted for 85% of cases in 2018 in Germany. Only 6.1% of reported syphilis cases were in women [8]. Co-infection with human immunodeficiency virus (HIV) was reported in 46% of all reports of syphilis in MSM in Germany; the proportion was significantly lower (6.7%) for a probable heterosexual route of infection [8]. Other reported co-infections in patients diagnosed with syphilis in Germany were chlamydia, gonococci, hepatitis B, and hepatitis C, all with significantly lower incidence (0.4 to 7% depending on the pathogen and the various risk groups) [9].

## Clinical manifestations of neurosyphilis

### Overview of clinical manifestations of syphilis

In the early stage of syphilis, primary syphilis, rough induration occurs at the entry point of the pathogen approximately 10 days to 3 months after infection. This results in a painless ulcer with regional lymphadenopathy. In 60–70% of all patients, the primary ulcer remains the only manifestation of syphilis [10].

In the phase of hematogenic and lymphogenic spread, one speaks of secondary syphilis. The symptoms vary: patients can suffer from fever, tiredness, headache, joint pain, or muscle pain [3, 11]. Hard swelling of many lymph nodes is almost always found. In addition, various rashes and enanthemas (syphilids) occur. Almost all organs can be affected; involvement of the central nervous system (CNS) is also not uncommon in this early phase [11]. With the help of the rabbit inoculation test (RIT), investigations revealed a pathogen in the cerebrospinal fluid (CSF) in 30% of those examined in the secondary stage of early syphilis; CSF pleocytosis (usually asymptomatic) was found at this stage in 40% of those examined [12]. Since only 5–10% of those affected develop neurosyphilis years to decades later in the natural course of syphilis (18), spontaneous “healing” in the CNS is obviously possible.

In the late phase (tertiary stage), many years after infection, there are tuberous skin changes, ulcerating granulomatous lesions in different organs (so-called gummas), and cardiovascular changes (mesaortitis, aneurysms). In the tertiary stage, even high-dose antibiotic treatment is not entirely successful; some effects of the disease persist.

### Neurosyphilis

Depending on the time since infection, early neurosyphilis is distinguished from late neurosyphilis. Early neurosyphilis primarily includes syphilitic meningitis and meningovascular neurosyphilis (although the latter can also occur many years after infection and is therefore often included as a manifestation of late neurosyphilis). Classical forms of late neurosyphilis are tabes dorsalis, paralytic neurosyphilis, and the appearance of syphilitic gummas.

#### Early neurosyphilis

##### Meningitis

Syphilitic meningitis is a manifestation of early neurosyphilis at the stage of secondary syphilis, usually within the first year after infection, but occasionally up to a few years later. Syphilitic meningitis is characterized by headache, meningism, nausea/vomiting, and cranial nerve lesions (affecting one or more of the third, seventh, and eighth nerves); the optic nerve can also be involved. In addition, polyradicular symptoms and vascular brainstem syndromes can occur.

Ocular (posterior uveitis or panuveitis) and otological (hearing loss, vestibular failures) manifestations of syphilis are possible at any stage, but are found more frequently in patients with meningovascular neurosyphilis [13]. Ocular manifestations appear to be particularly common in HIV-positive persons and in asymptomatic neurosyphilis [14].

##### Meningovascular neurosyphilis

Meningovascular neurosyphilis is a form with vascular and meningeal manifestations. In the literature, meningovascular syphilis is variably classified as early neurosyphilis or late neurosyphilis because it may occur both a few months after infection and some years later. It is now one of the most common manifestations of neurosyphilis. The risk of stroke is increased by a vascular manifestation of syphilis. The symptoms of the vasculitic component are clinically diverse, depending on the localization. Neurosyphilis should be considered in young, especially male, patients who have a stroke despite the absence of a vascular risk profile. The meningeal component manifests itself as headache, cranial nerve lesions, optic damage, and, rarely, hydrocephalus.

##### Syphilitic gummas

Syphilitic gummas, a rare manifestation, are circumscribed granulomas that develop from the meninges of the cerebral convexity [15–17]. Depending on the location, they are clinically silent or cause focal neurological deficits, hydrocephalus, or epileptic seizures. Like meningovascular neurosyphilis, syphilitic gummas can also appear in the late stage.

#### Late neurosyphilis

##### Tabes dorsalis

Tabes dorsalis is a chronically progressive dorsal radiculoganglionitis with loss of reflexes in the lower extremities, pallanesthesia, pupil disorders, hyperextensibility of the knee and hip joints, micturition disorders due to deacidification, and optic damage. The patients complain above all of stabbing pain that radiates into the legs or the abdomen (so-called tabular crises).

##### General paresis

General paresis is a chronic progressive encephalitis. The typical symptoms are increasing cognitive deficits, weakness of discrimination and judgment, psychotic episodes, speech disorders, headache and dizziness, abnormal pupillary reaction, tremor of the tongue, facial tremor, epileptic seizures, reflex anomalies, and finally severe dementia, urinary and fecal incontinence, and marasmus [18].

#### Other manifestations of neurosyphilis

##### Asymptomatic neurosyphilis

Asymptomatic neurosyphilis is diagnosed when syphilis serology is positive, lymphocytic pleocytosis and protein elevation are found in the CSF, and/or a positive Venereal Disease Research Laboratory (VDRL) test in the cerebrospinal fluid is detected in the absence of clinical symptoms [3].

##### Atypical manifestations

Cases of neurosyphilis were published many years ago that could not be classified as syphilitic meningitis, meningovascular neurosyphilis, tabes dorsalis, general paralysis, or syphilitic gummas [19]. Such cases were referred to as “modified neurosyphilis”, “formes frustes”, or “lues liquorpositiva tarda”. The more recent literature includes cases of temporal lobe encephalitis that resembled herpes simplex virus 1 (HSV-1) infection or limbic encephalitis [20–22]. The main clinical manifestations are cognitive deficits and epileptic seizures. In addition, other structures such as the thalamus, the parietal lobe, and the occipital lobe may be affected. Ultimately, these cases seem most likely to represent mixtures of different classic forms, such as meningovascular neurosyphilis and general paresis.

## Diagnosis

### Overview of tests for syphilis and neurosyphilis

#### Background

Several test systems are available for diagnosing syphilis. A distinction is made between direct and indirect methods of pathogen detection.

*Direct detection* of the pathogen can be carried out using dark-field microscopy and polymerase chain reaction (PCR). However, due to the low pathogen density in the cerebrospinal fluid, both of these methods are usually of little or no assistance in neurosyphilis. The use of PCR for the detection of *T. pallidum* in the CSF for the diagnosis or exclusion of neurosyphilis cannot be recommended due to its low sensitivity (31.7 to 63.7%) and insufficient specificity (39.9 to 98%) [23, 24].

Serological tests are available for an *indirect detection* of the pathogen. A distinction is made between non-treponeme-specific antibody tests and *T. pallidum*-specific antibody tests. The non-treponeme-specific antibody tests include the VDRL test and the rapid plasma reagin (RPR) test. Both of these tests detect antibodies against cardiolipin, which is present not only in the cell wall of *T. pallidum*, but also in the mitochondria of human, animal, and plant cells. In syphilis, cardiolipin antibodies are an important, albeit not very specific, marker for the activity of the disease. In *T. pallidum*-specific antibody tests, antibodies against *T. pallidum* are detected using different methods; however, cross-reactions with other *Treponema* spp. and other spirochetes (especially *Borrelia* spp.) are possible. The *T. pallidum* particle agglutination (TPPA) test, the *T. pallidum* hemagglutination test (TPHA), the *T. pallidum* latex agglutination (TPLA) test, the fluorescent treponemal antibody absorption (FTA-ABS) test and the detection of IgG and/or IgM antibodies directed against *T. pallidum* using enzyme-linked immunosorbent assay (ELISA) or Western blot are available. In early phases of syphilis, IgM antibodies are detected first. The sensitivity of a positive *T. pallidum*-specific antibody test is generally high; however, a positive test says little about the disease activity, since antibodies can persist for life after adequate treatment or spontaneous healing of syphilis.

Especially in neurosyphilis, the uncertainty of the classic tests makes it difficult to arrive at the diagnosis. In most regions of the world, the diagnostic criterion for (active) neurosyphilis is a reactive CSF VDRL test together with an elevated CSF leukocyte count (> 5 cells/μl) and high CSF protein (> 40 mg/dL) [25]. The specificity of the VDRL test in the CSF is high, particularly in late stages of neurosyphilis (up to 100%) [13]. However, the test shows sensitivity of only 30 to 85.7% [26–28], depending on the stage of neurosyphilis; a negative CSF VDRL test hence does not rule out neurosyphilis. In contrast, the sensitivity of a positive CSF/serum TPPA index is relatively high [29]. However, this cannot be used as an activity parameter: even if the infection has been cleared (by antibiotic therapy or spontaneously), a high TPHA, TTPA, or TPPA CSF/serum antibody index can be found over a period of years or even decades, and both the TPHA test and the TPPA test can yield false-positive results in patients with Lyme disease.

A potentially interesting new CSF marker in patients with neurosyphilis is the chemokine CXCL13, which is significantly elevated in the CSF of patients with neurosyphilis [30–32]. Increased CXCL13 levels do not appear to be present in all patients with neurosyphilis, however, and different CSF levels of CXCL13 in patients with symptomatic neurosyphilis and asymptomatic neurosyphilis have been reported [31]. It also remains unclear to what extent CXCL13 might be helpful for diagnostic differentiation of neurosyphilis from other diseases, since CSF restricted CXCL13 levels are also found with other B cell-mediated CNS diseases (e.g. neuroborreliosis and CNS lymphoma) [33]**.** In addition, CXCL13 could be a helpful parameter for assessing a therapeutic effect, since its concentration in the CSF decreases rapidly after successful treatment [31]. Currently, the determination of CXCL13 in the CSF in neurosyphilis is not recommended due to the lack of large studies.

#### Recommendation

The determination of CXCL13 in the CSF in neurosyphilis is not recommended due to the lack of large studies.

### Diagnostic criteria

#### Background

Because of the uncertainty of the non-treponeme-specific antibody tests, modified diagnostic criteria for neurosyphilis have been chosen in German-speaking countries. In principle, the diagnosis of neurosyphilis is made from a combination of (1) clinical findings, (2) CSF parameters, and (3) the detection of intrathecal synthesis of antibodies against treponemes.

#### Recommendation

A patient suffers from *probable neurosyphilis* if point 4 and any two of points 1 to 3 and always point iv are positive [5]:

(i) Subacute or chronic progressive course of neurological and/or psychiatric symptoms with phases of deterioration and partial remission
(ii) Pathological CSF findings with mixed-cell or mononuclear pleocytosis or blood–CSF barrier damage or IgG-dominant immune reaction in the CNS
(iii) Favorable influence on the course of the disease and/or CSF findings (especially pleocytosis) exerted by antibiotics recommended for the treatment of neurosyphilis
(iv) Positivity of the TPHA (or TPPA) test and the FTA-ABS test in the serum.

A patient is suffering from *proven neurosyphilis* if, in addition to fulfillment of the criteria of probable neurosyphilis, a local treponeme-specific antibody reaction can be detected, measured via the detection of intrathecal production of antibodies against *T. pallidum* (ITpA = intrathecally produced *T. pallidum* antibodies) or a TPHA CSF/serum antibody index. If at the same time the VDRL test in the CSF is positive, the diagnosis is *confirmed*.

### Work-up in suspected neurosyphilis

#### Background

**Fig. 1:**
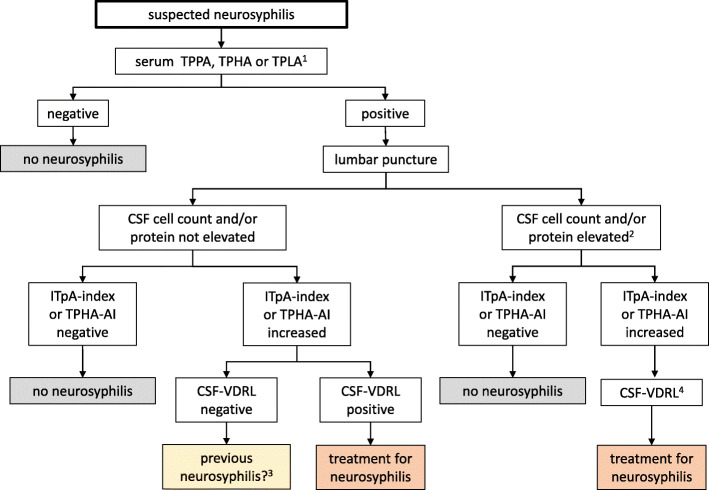
Work-up in patients with suspected neurosyphilis. (1) Confirmation of a positive test using a confirmatory test is recommended (see text). (2) In patients with HIV, a discrete disruption of the blood–brain barrier may occasionally be seen. Also, a slightly increased cell count can be the result of the HIV infection itself. (3) If suspicion of neurosyphilis persists, treatment should be considered. (4) The VDRL test is recommended to create a basis for future follow up investigations. For details see text. TPPA = *Treponema pallidum* agglutination test; TPHA = *T. pallidum* hemagglutination test; TPLA = *T. pallidum* latex agglutination test, ITpA = intrathecal *T. pallidum* antibodies; AI = CSF/serum antibody index; VDRL = Venereal Disease Research Laboratory

Typically, neurosyphilis shows an increase in the number of cells and blood–CSF barrier disruption (Fig. 1), reflected in increased CSF protein levels. In 110 patients with symptomatic neurosyphilis and 154 patients with asymptomatic neurosyphilis, the CSF cell count was found to be increased in 82.7 and 81.2% of cases, respectively, and the CSF protein in 68.1% versus 28.6% [34]. In the case of simultaneous HIV infection, pleocytosis was found in only 58.7% of 92 patients, while CSF protein was elevated in 53.3% [14]. However, the significance of small increases in CSF cell count in patients with HIV is limited with regard to the diagnosis of neurosyphilis, as pleocytosis is often found in patients whose only infection is HIV [3]. The changes in the CSF depend on the manifestation of neurosyphilis. In meningitic neurosyphilis, there is often an increase in the number of cells, although this does not appear to be the case in later stages [14]. In meningovascular neurosyphilis, CSF protein is particularly high [14]. Of note, an increased total CSF protein is a non-specific parameter. It is therefore advantageous, though also not very specific, to consider the blood–CSF barrier function as determined by the CSF to serum ratio of albumin (QAlb) (according to Reiber) when evaluating the activity of the disease [35].

#### Recommendation

If neurosyphilis is suspected clinically, a syphilis test should first be carried out in serum (Fig. 1) using TPPA, TPHA, or TPLA. Positive results should be confirmed using the FTA-ABS test or through antibody detection by ELISA. IgM antibodies can be negative in the late stages of neurosyphilis. Detection of antibodies in the serum alone does not confirm the diagnosis, because antibodies from a previous episode of syphilis can persist for the patient’s lifetime. In the case of a positive *T. pallidum*-specific antibody response, the next important step is examination of the CSF. The main CSF parameters are cell count, protein, lactate and/or CSF/serum glucose ratio, measurement of CSF proteins according to Reiber (albumin quotient, IgG, IgA, and IgM ratio), and the determination of a CSF/serum IgG antibody index for specific antibodies against *T. pallidum* (ITpA or TPHA CSF/serum antibody index).

#### Recommendation

In HIV-negative patients with increased CSF cell counts and/or blood-CSF barrier damage and an increased ITpA or TPHA CSF/serum antibody index, treatment for neurosyphilis should be considered. If intrathecal antibody production is found in the absence of any other CSF changes (cell count or protein increase), it is often unclear whether the elevated titer is the residue of expired or treated neurosyphilis or the sign of a relevant infection. In the case of a positive CSF VDRL test, treatment should be carried out as for confirmed neurosyphilis [3]. Treatment should also be considered if clinical symptoms cannot be explained in any other way when intrathecal antibody production (ITpA) is found in the absence of further parameters of activity (increased cell count, increased protein, or a positive CSF VDRL test) and the patient has never been treated with antibiotics directed against neurosyphilis.

#### Recommendation

If treatment is considered (Fig. 1), IgM determination and VDRL testing in the serum and in the CSF should always be performed to form the basis for a later assessment of the course of the disease.

### Work-up in possible latent asymptomatic neurosyphilis

#### Recommendation

In the following constellations, CSF analysis is indicated in the case of a positive treponeme-specific seroreaction:

- Neurological (e.g. epileptic seizures, focal neurological deficits), psychiatric (e.g. changes in personality/behavior), ophthalmological (e.g. papillitis, uveitis), or otological (e.g. hearing impairment) symptoms
- Clinical signs of gummas or cardiovascular manifestation of tertiary syphilis

#### Background

Patients with HIV are at increased risk of latent asymptomatic neurosyphilis, especially if they have not received antiretroviral therapy and the HIV viral load is high [36, 37]. In HIV patients with latent asymptomatic neurosyphilis the CD4 cell count in is usually reduced [36]. In a study by Wang et al. the CD4 cell count was < 200/μl in most patients with latent asymptomatic neurosyphilis [14]. In patients with proven syphilis and HIV co-infection, CSF examination should also be considered in the event of a positive treponeme-specific seroreaction if advanced immunodeficiency (cell count < 200/μl) is present [5].

#### Recommendation

The following criteria can be used pragmatically to determine whether lumbar puncture is indicated (that is the case if at least two out of four are met):

- CD4 cell count ≤ 200 cells/μl
- Untreated HIV infection
- Detectable HIV load
- High VDRL titer (> 1:64)

#### Recommendation

Treatment against neurosyphilis should be started in the case of positive treponeme-specific intrathecal antibody production and a positive VDRL titer in the CSF. If syphilis treatment has been carried out in advance, a positive CSF VDRL titer should be interpreted by taking into account titer levels in previous CSF specimens). In asymptomatic HIV-positive patients with positive treponeme-specific intrathecal antibody production but a negative VDRL test in the CSF, treatment for neurosyphilis can be considered if no course of treatment for syphilis has been completed before. However, in such cases, broad differential diagnosis of any CSF pleocytosis is essential.

## Treatment

### Antibiotic therapy

#### Background

The treatment of choice for neurosyphilis is intravenously administered penicillin G at a dose of 4 × 6 million IU per day, 5 × 5 million IU per day, or 3 × 10 million IU per day (corresponding to 3–4 million IU every 4 h) for 14 days (at least 10 days) [3, 5, 25, 38, 39]. This scheme is used for symptomatic and asymptomatic neurosyphilis, as well as for all forms of syphilis with HIV co-infection. Since an increased rate of epileptic seizures was observed with the administration of high-dose penicillin G [40], adjuvant anticonvulsive treatment should be given to patients with previous epileptic seizures or epilepsy-typical potentials on EEG.

An alternative therapeutic regimen for patients with suspected or confirmed syphilitic CNS involvement is intravenous administration of 2 g ceftriaxone per day (2 g every 24 h) for 14 days [25, 41]. There is insufficient evidence for a possible benefit of an initial dose of 4 g as recommended in previous versions of the guideline.

Treatment with doxycycline (2 × 200 mg per day [200 mg every 12 h] for 28 days) can be considered as an alternative. The rationale for this treatment approach is the well-known effectiveness (and recommendation) of doxycycline therapy for systemic forms of syphilis [3, 38], sufficient penetration of doxycycline into the CSF at a dose of 2 × 200 mg per day [42], and several case reports of positive treatment results with doxycycline in neurosyphilis [42–44]. Owing to the lack of randomized studies, however, there is no recommendation from the CDC or the European guideline for the treatment of neurosyphilis with doxycycline [3, 25]. Tetracyclines are contraindicated in pregnant women and in children up to 8 years of age because they cause yellowing of the teeth.

#### Recommendation

Neurosyphilis should be treated with high dosages of intravenous penicillin G or ceftriaxone. Antibiotic therapy is indicated at all stages of neurosyphilis.

### Treatment of complications

#### Background

One important complication of neurosyphilis treatment is the occurrence of a Jarisch–Herxheimer reaction. The presence of this reaction must be considered if general symptoms such as fever, headache or muscle pain, fatigue, tachycardia, increase or decrease in blood pressure, leukocytosis, and relative lymphopenia, as well as seizures, occur 12–24 h after the start of antibiotic treatment. The Jarisch-Herxheimer reaction is common in the secondary stage of early syphilis, but has been observed in only 1–2% of cases of neurosyphilis. Affected patients should be monitored and treated symptomatically with non-steroidal anti-inflammatory drugs [3]. Antibiotic administration should not be interrupted. Although adjuvant administration of steroids in the treatment of neurosyphilis or of a Jarisch-Herxheimer reaction has been discussed repeatedly in case reports, there are no studies that support this policy [45, 46].

#### Recommendation

The routine use of corticosteroids is not recommended in the treatment of neurosyphilis.

## Follow-up

The successful treatment of neurosyphilis is primarily assessed by the clinical response and the improvement of CSF abnormalities. If the clinical response to treatment is good, a follow-up examination should be carried out around 3 to 6 months after treatment. A decrease in the number of CSF cells and CSF protein levels are then to be expected [47]. Subsequently, the CSF should be checked every 6 months until the CSF cell count has normalized (which usually occurs within 2 years after successful treatment).

A decline in serum IgM antibody kinetics within 6–12 months can also be helpful for assessing the course. Disappearance of the treponeme-specific IgM antibodies is usually observed within 18 months. In the event of reinfection, or if there is a long interval between infection and the start of treatment, treponeme-specific IgM antibodies can remain in the serum for longer. A reduction in the CSF VDRL titer by three to four dilutions is also often observed within the first year; a negative VDRL test after treatment is considered to be evidence of successful treatment [47]. In the event of reinfection or a long interval between infection and the start of treatment, lipoid antibodies can remain detectable for an extended period, but their level should nevertheless drop with effective therapy. The TPPA and FTA-ABS tests, like the ITpA index, are unsuitable for treatment control because they usually remain positive for life.

If there is no decrease in the CSF cell count within 6 months, if the CSF cell count continues to increase 2 years after treatment, or if there is a significant (re-)increase in a non-treponeme-specific test by a factor of 4, an alternative antibiotic therapy should be considered. In patients with simultaneous HIV infection, the CSF changes regress more slowly [47]. If there is an isolated persistent increase in the number of cells despite adequate therapy, the presence of another disease should be investigated.

## Prognosis

The prognosis of neurosyphilis has improved significantly in recent decades due to rapid and adequate antibiotic treatment. While 586 patients died of syphilis in the USA in 1968, between 1998 and 2015, 24 to 46 deaths from syphilis per year were reported [48]. Residual neurological symptoms are common in patients with neurosyphilis. In a retrospective study of 142 patients with neurosyphilis, sequelae were found in 41.8% of the patients: cognitive deficits (28.8%) such as memory disorders (10.1%), signs of damage to the first motor neuron (18.6%), seizures and ataxia (16.9% each), cranial nerve palsy (15.2%), visual disturbances (11.8%), and gait disorders, aphasia. and hemiparesis/hemiplegia (6.7% each) [49].

## Data Availability

Not applicable.
